# Embedding Researchers into Local Government Public Health Teams: Exploring Co-Design, Implementation and Evaluation Challenges Through Document and Contextual Analysis

**DOI:** 10.1007/s43477-025-00179-1

**Published:** 2025-09-03

**Authors:** Dylan Kneale, Rachael C Edwards, Claire Stansfield, Sarah Lester, James Thomas

**Affiliations:** EPPI Centre, UCL Social Research Institute, University College London, London, UK

**Keywords:** Embedded researchers, Evaluation metrics, Document analysis, Public health decision-making

## Abstract

Research suggests that evidence could play a greater role in local public health decision-making than is currently the case. Embedded researchers, located within policy and practice organisations while maintaining affiliations with research organisations, could represent a potential catalyst for bridging the gap between public health evidence generators and evidence users. Evidence examining this potentially transformational model of working is in its infancy. This study explored how the set-up of these roles influences their aims, features of their design, and their expected and observed outcomes, drawing on data from a scheme implemented in 23 Local Authorities (local government) in England. We analysed embedded researcher job descriptions, interviewed stakeholders, examined socio-economic contexts of organisations hosting embedded researchers, and examined publication and funded research data. Our findings indicate diversity in the role specifications, aims, and outputs of embedded researcher positions even within a single scheme. Despite the transformative potential of embedded researchers, challenges such as unrealistic expectations and inadequate evaluation metrics persist. Our analysis suggests that while Local Authorities hosting embedded researchers demonstrated some signs of increased research activity, there was considerable uncertainty as to whether these changes could be directly attributed to an embedded researcher. Co-design of roles between research and practice organisations increases the likelihood that embedded researcher roles were developed with a cogent set of aims, and that these reflect the needs of all organisations involved. The results attest to the complexities of evaluating embedded researcher roles and emphasise the need for tailored, context-sensitive approaches to understanding the contributions of embedded researchers.

**Supplementary Information:**

The online version contains supplementary material available at 10.1007/s43477-025-00179-1.

It is now over a decade since the transformation of the National Health Service in England saw public health ‘return home’ to Local Authorities (Gorsky et al., 2014), who are now responsible for commissioning across most aspects of public health services delivered locally (Kneale et al., 2017). While this transition was initially accompanied by continuity in funding levels, public health teams subsequently faced real terms cuts in funding allocations (Buck, 2018; Kneale et al., 2019), and more recently were at the forefront of the response to the COVID-19 pandemic. One of the many challenges of these shifts in the context of decision-making was in understanding evidence needs, and how these may have changed to reflect a new and more (locally) politicised environment that was facing budgetary cuts. Many reviews of public health decision-making have been suggestive of a long-standing pattern of underutilisation of research evidence (Dam et al., 2023; Kneale et al., 2017; Orton et al., 2011).

Document analyses conducted after the transition of responsibilities highlighted that Local Authorities were more successful at sourcing and applying evidence of need, rather than identifying evidence of effectiveness (Beenstock et al., 2014); and that there were strong preferences for particular forms of quantitative evidence and less around systematic review or qualitative evidence (Kneale et al., 2018). Further primary research with decision-makers indicated that the politicised nature of decision-making within Local Authorities had increased the importance for research evidence to demonstrate its ‘local credentials’ (Kneale et al., 2019), and that research evidence often addressed much narrower research questions than the broad policy-level questions that decision-makers faced and needed support with (Kneale et al., 2019; South & Lorenc, 2020).

## Embedded Researchers as a Catalyst To Stimulate Research Activity

Identifying ways of stimulating a more regular dialogue between evidence generators/synthesisers and evidence users could help to promote the utilisation of research evidence in public health. These could include new transformational ways of working and developing relationships across boundaries. Others have described the environment in which public health research is generated as seemingly detached from the ‘messy’ and ‘complex’ decision-making worlds (Cheetham et al., 2023). The intervention of interest in this paper is that of embedded research, which could enhance the connections and interactions between evidence generators and evidence users, and help researchers make sense of the complexity faced by decision-makers. Embedded researchers within local public health decision-making contexts tend to be those ‘researchers who work inside host organisations as members of staff, while also maintaining an affiliation with an academic institution’ (Vindrola-Padros et al., 2017). Embedded researchers may have the potential to address the issues outlined above in a number of ways including through (i) actively researching and contributing to decision-making processes; (ii) changing cultures around research engagement, direct research utilisation, or research generation; and (iii) may help to produce research evidence that more closely matches the need of decision-makers with regards to questions asked and contextual salience (Kneale et al., 2024).

### Complexities in the Implementation of Embedded Researchers

While embedded researchers may hold promise in bridging the divide between research generators and research users, and is a model of working that is rapidly expanding within the UK public health context, this paper investigates three interrelated issues relating to the deployment of embedded researchers: (i) the expectations of the roles; (ii) the extent to which variable levels of co-design may shape embedded researcher roles and their expectations; (iii) the extent to which the far reaching expectations of embedded researchers’ roles can be measured and the implications for evaluating these posts.

A factor that adds significant complexity to the effective design and evaluation of embedded researcher roles is their dual affiliation with research and practice organisations. These institutions are often highly disparate, with differing measures of success, processes, and systems. For example, Marshall et al. (2014) describe how embedded researchers “*need to understand the institutional drivers of different partners—peer reviewed publications*,* grant income and research studentships might be vital for universities*,* but demonstrable local population health improvement*,* financial balance and target achievement would be goals for the health sector*” (p804). These cross-organisational tensions can create challenges for embedded researchers, including for their own career progression (Coates & Mickan, 2020; Vindrola-Padros et al., 2018). Here we also view indicators such as peer-reviewed publications and grant income as examples of instrumental indicators of the influence of embedded researchers – indicators of change that can be collected through common standardised measures.

While prior work has explored embedded researchers’ experiences of dual affiliation, little research has investigated organisational collaboration in the set-up of embedded researcher roles and how the involvement of multiple, diverse organisations, and their power relations, could affect the design of embedded researcher schemes. Further investigation into such contextual influencers can help us to understand the extent to which embedded researcher roles are responding to local priorities and needs.

With respect to the challenges of evaluating embedded researcher roles, gaining an accurate understanding can be hindered by unrealistic expectations (Kneale et al., 2023). For example, within frameworks published to support the design of embedded researcher interventions, the intended aims are often far-reaching and include changing the capacity of an organisation to produce knowledge and even around its capacity to ‘deliver services and to generate income’ (Ward et al., 2021, p762). With regards to organisational capacity, others have noted that the aims of embedded researcher interventions are two-fold in that they are expected to (i) undertake or lead their own research and (ii) to build the capacity of others within host teams to undertake research (Mickan & Coates, 2022). However, the capacity of embedded researchers to undertake such widescale organisational transformation is unclear, with many interventions being conducted by a single embedded researcher (for example Cheetham et al. (2018). Given these far-reaching expectations (particularly in relation to capacity building), it is unsurprising that embedded researcher roles also often lack clear evaluative approaches (Ward, Tooman, Reid, Davies, & Marshall, 2021). For example, in a survey with embedded researchers in the Australian healthcare sector, almost a quarter of participants indicated that they were unsure of their key performance indicators or did not have specific measures of success (Mickan & Coates, 2022).

The research here seeks to respond to these gaps through exploring what is expected of embedded researcher roles; how collaboration, or lack thereof, in the set-up of embedded researcher roles affects the aims and expectations of the posts and their implementation; and to illuminate the challenges of evaluating embedded researcher posts.

## Study Design

We undertook a multi-methods exploration of a particular scheme of embedded researchers developed by the UK’s National Institute for Health and Care Research (NIHR; referred to as the national research funding agency in places). The NIHR is the largest funder of clinical health, public health and social care research in the UK with a mission to improve the nation’s health. Its delivery in England is coordinated through regional networks, with each region covering multiple Local Authorities (a form of local or municipal government) areas. The exploratory scheme considered here saw researchers – known as Public Health Local Authority Research Practitioners (PHLARPs) - embedded across twenty-three diverse Local Authority public health settings in England (with some being embedded across multiple settings and with multiple posts being supported through job-sharing arrangements in others). We refer to PHLARPs as ‘embedded researchers’ in the remainder of the manuscript. The first two embedded researchers started in post in 2020 and most of the remaining cohort followed in spring 2021. As such, in this study, Local Authorities served as the embedded researchers’ affiliated practice organisations while the national research funding agency functioned as their affiliated research organisation who were also responsible for initiating and managing the overarching program (e.g., through recruiting Local Authorities). In many cases, the embedded researchers were affiliated with both the national research funding agency and a university partner while embedded in a Local Authority. The intention of the initiative was to facilitate and enhance public health cultures of research activity within local government. Although the embedded researcher roles were connected by overarching aims, they were meant to be operationalised with some flexibility across Local Authorities. As such, this intervention provided an opportunity to explore diverse embedded researcher roles influenced by complex layers of management arrangements involving both research and practice organisations.

The study is guided by four research questions (RQs):

1. *How do organisations collaborate in the set-up of embedded researcher posts?*
2. *How do the underlying aims and expectations of embedded researcher posts vary?*
3. *How do contextual factors shape embedded researcher roles?*
4. *How can we measure the influence of embedded researchers and do instrumental indicators of research activity help us understand their contributions?*

Collectively, these research questions provide a multidimensional exploration of the expectations surrounding embedded researcher positions, the ways in which collaboration across research and practice organisations can influence the expectations of these posts, and the extent to which expectations of embedded researchers’ roles can be measured. This study is part of a wider programme of research on this embedded researcher scheme that is reported in detail elsewhere (Kneale et al., 2023).

### Study Participants

This study draws on interviews with NIHR-affiliated stakeholders involved with the set-up and implementation of the embedded researcher scheme as well as secondary data sources. This study involved seven stakeholders who were responsible for overseeing and creating the PHLARP scheme as a whole and/or were involved in the creation and design of individual posts (further data are not presented on the characteristics of these individuals to maintain anonymity).

### Ethics Approval

Ethical approval was granted from the UCL Institute of Education’s Research Ethics Committee (REC1540).

## Methods

### Interviews

Interview data contributed to addressing all RQs. All interviews were conducted remotely using Microsoft Teams or Zoom and were transcribed in full; the interviews lasted between approximately 25 and 50 min. Interviews were semi-structured, with an interview schedule developed to understand broadly: (i) what stakeholders understood to be the aims of the embedded researcher posts and how the expectations/aims of the posts were identified; (ii) the way in which Local Authorities collaborated in creating the posts and the process of recruiting embedded researchers; (iii) and the broad reflections of stakeholders on the implementation of the embedded researcher posts, including in relation to co-design and evaluation. At the time of the interviews, the embedded researchers had been in post between 6 and 10 months.

### Review of Documents

#### Job Descriptions

To analyse the aims of the embedded researcher scheme (addressing RQ 2), we obtained 16 job descriptions for the embedded researcher posts that were made available to the team by the funder, as well as those sourced through independent internet searches. To help preserve the anonymity of the participants involved in the research, we do not include details around the Local Authority when quoting from job descriptions.

#### Contextual Data

To examine the types of Local Authorities that researchers were embedded into (addressing RQ 3), we draw on data from the Office for Health Improvement and Disparities (Office for Health Improvement and Disparities, 2023) to examine if differences exist in the social and health profile of those that hosted embedded researchers and those that did not. We focussed on indicators around poverty, health and healthy lifestyles that included the level of deprivation (index of multiple deprivation score), an index of inequality in life expectancy at birth for males and females, and the percentage of adults classified as obese.

#### Measures of Research Activity

To explore the impact of embedded researchers on increasing research activity (RQ 4), we considered three ‘instrumental’ indicators that reflect research facilitation, research funding, and research publication respectively. These are termed instrumental indicators as they represent standardised and common measures of research impact within academia, and we were interested in their relevance to embedded researcher roles in the context of public health. Firstly, we explored the extent to which Local Authorities who hosted embedded researchers became study sites on the NIHR Portfolio of studies compared with those that did not. For Local Authorities, registering studies on the national research funding agency’s portfolio resulted in access to methodological advice, support, and training. Secondly, we explored whether sites where embedded researchers were placed were more successful in obtaining (further) research funding from the national research funding agency. We used web-scraping approaches (Bradley & James, 2019), implemented through R, to gather data from the NIHR website on the extent to which the contracting organisation (a Local Authority) corresponded with one of the Local Authorities that hosted an embedded researcher. The data were collected in March/April 2023. Given that in practice very few Local Authorities had obtained national research funding agency funding, these data were analysed descriptively. We also augmented these data with data on Health Determinants Research Collaboration (HDRC) funding decisions which were announced for thirteen Local Authorities (twelve in England), but were not (yet) included on the main NIHR funding database (NIHR, 2022). HDRCs have parallels with the aims of embedded researchers in that they are intended to ‘embed a culture of evidence-based decision-making’ into Local Authorities. The data did not allow us to examine where a Local Authority may have submitted an unsuccessful proposal for a HDRC or other type of funding, which also signals engagement with research systems. Finally, we conducted further web-scraping over March/April 2023 and automated searching of the PubMed database to provide a snapshot of the extent to which authors based in, or with an affiliation with, Local Authorities were authors of peer-reviewed studies in public health. Using the full name (e.g. Derby City Council) or a contracted/simplified version (e.g. Derby Council), we ran automated searches through R to create a data frame that included the number of publications with a Local Authority affiliated author by year, using ‘Public Health’ as an additional free-text limiter.

### Data Analysis

#### Interviews

Thematic analysis was used to analyse the interviews. Specifically, we used template analysis as a way of understanding the findings, which involved developing a coding template based on an initial transcript and applying this to subsequent transcripts (Brooks et al., 2015). The coding template, which involved creating nodes and sub-nodes within NVivo 20 (QSR International Pty Ltd., 2020), was then revised and reapplied with additional transcripts (King, 2012). The interviews were coded by the main author with the second author conducting checks on a 25% sample of each. As there was a strong level of agreement, double coding all interviews was deemed unnecessary.

#### Documents

Job descriptions were coded using EPPI-Reviewer (see Thomas et al., 2020) by the main author with a second author (RE) conducting checks on a 25% sample. The results are summarised descriptively and narratively, based on tabulations of characteristics (e.g., expected duties, desired experience), as well as patterns and typologies based on these patterns (e.g., whether the aims were focused predominantly on Local Authority concerns, NIHR concerns, or both).

Contextual data on Local Authorities were employed to explore how the characteristics of Local Authorities who hosted embedded researchers differed from those that did not through descriptive analyses. To allocate embedded researchers to specific Local Authorities in which they were embedded we drew both on contact lists as well as descriptions from interviews that indicated which Local Authorities they worked most closely with and effectively became embedded. In several cases, embedded researchers worked across a small number of Local Authorities closely (typically no more than two) and in these cases both named areas were considered as having hosted embedded researchers. Using these approaches, we identified 23 Local Authorities in England as having hosted embedded researchers through PHLARP scheme of interest (out of 152 in total with public health responsibilities).

Measures of research activity were analysed descriptively for all indicators initially. For data on publication trends, we constructed bivariate zero-inflated negative binomial regression models (Kirkwood & Sterne, 2010) to examine whether authors that were based in, or affiliated with, Local Authorities that hosted an embedded researcher were more or less likely to have been authors or co-authors of peer reviewed studies in Public Health, with zero-inflated models developed to reflect the excess of ‘zero’ publication counts across several Local Authorities. Finally, to better account for unobserved heterogeneity, we created longitudinal models akin to a difference-in-difference model (Wooldridge, 2015) examining publication trends for the three years preceding the implementation of the embedded researcher scheme and the two complete calendar years during and following their implementation (2021 and 2022); we created linear and binary specifications to better account for the high number of zero counts in the publication data.

## Results

### Summary of Interview Themes

The supplementary materials provide an overview of all the main themes that emerged within the interviews. Findings with respect to challenges in collaboration and implementation are discussed directly below, and we also elaborate on findings with respect to the theme of setting and evaluating expectations and aims for embedded researchers.

#### Challenges in Collaboration in Designing Embedded Researcher Posts

Overwhelmingly, stakeholders described their experiences of how the roles were designed and implemented in the context of the COVID-19 pandemic. COVID-19 raised challenges in terms of recruitment, and some stakeholders described anecdotal conversations with Local Authorities where they were told to ‘forget it’ if they expected participation during the pandemic, which led to delays and protracted periods of recruitment. Stakeholders discussed that the pandemic meant that roles, which had been intended to be in-person, were implemented remotely and expressed concern about the implications for the wellbeing of embedded researchers. In one case however, the COVID-19 pandemic was also viewed as having raised the profile of research in general which subsequently piqued an interest among Local Authorities for embedded researcher and similar roles; this was not, however, a widely held belief among the stakeholders:

I think research now… the whole country understands it a little bit better because of Covid and the vaccine and what it’s all about. Suddenly everybody’s like “oh research, yeah!”. You talked about research pre Covid it was like “Uhhhh. What’s that about? Research into what?*”.* People have a better understanding of it now, but at the time we were going out for the posts, nobody knew what it was about, what are you going to get them to do.

While the COVID-19 pandemic was identified as a significant barrier to recruitment, barriers were also attributed to variable relationships between the NIHR and Local Authorities, with some Local Authorities being described as ‘quite engaged’ and others said to have ‘absolutely no contact at all’:

I think one of the things was some people really struggled to fill the post. It was a bit of a mixed bag. Some places filled the posts quite quickly, and some really struggled. So, they either got no one to interview, or when they did interview, no one was appropriate. I think we had a bit of variation.

In addition to the barriers to recruitment, some stakeholders expressed concern that there could be barriers to retention of individual embedded researchers given that the sustainability of the roles was uncertain. All roles were offered on a limited fixed-term basis, and in one case an embedded researcher had left the role after less than a year due to their concerns about the sustainability of their post.

#### Aims of Embedded Researcher Roles

All stakeholders reflected that the creation of the embedded researcher posts was intended to be flexible and responsive to the needs of Local Authorities. Some also reflected that this flexibility allowed the local NIHR and Local Authority to work collaboratively: “Because each of them had applied for a different focus and therefore we made sure that actually the role description allowed that to be part of the appointment in the end. Because I think that’s quite important, that they own it. It’s their role and it’s their vision.”

The analysis of job descriptions (see below) indicates that embedded researcher roles appear, in most cases, to be designed around an ambition to expand the portfolio of public health studies supported by the national research funding agency. Indeed, those who had worked on designing, managing, or recruiting the roles, all of whom had close connections with the NIHR itself, often expressed views that a primary aim of creating embedded researcher posts was to enhance national research funding agency support to Local Authority public health research. NIHR support for research was viewed as a way of enhancing the quality of research being conducted in Local Authorities. Creation of embedded researcher posts was viewed as a reflection of NIHR’s expanding remit into public health research, which in turn required a diversification both in terms of the type of research being undertaken and in terms of the teams leading research. Embedded researchers were viewed as a way for the NIHR to learn about how research was conducted within Local Authorities as well as to provide an inroad to higher quality research through adoption onto the NIHR portfolio:

And what you will find with Local Authorities is, there is research happening. If they’re linked to a university, it may be portfolio adopted, but if they’re not, it won’t be on the portfolio. So, a lot of the time that [Local Authority research] goes under our radar, we don’t know it’s happening. So, this is all part of what we need to learn. What are they doing? How are they doing it? How can we support them in doing that? And how can we then grow that?

There was also an assumption held by some that Local Authority research practices were underdeveloped and that the roles would help to bolster research cultures within Local Authorities. However, stakeholders tended to discuss enhancing research capacity in non-specific terms. No stakeholder provided examples of the challenges that Local Authorities they were engaged with faced in conducting or sourcing research and evidence. In addition, all stakeholders framed the work of embedded researchers in terms of enhancing research cultures, rather than other allied terms for example evidence informed decision-making:

In terms of starting the journey of embedding a culture, I think we’re starting on low base probably.

As part of the initiative, we’ll see by adding someone in there you’re increasing that capacity.

So, the aims were, particularly, it is about growing public health research. Growing an infrastructure for public health research, primarily, that is the aim.

#### Provision of Oversight and Support

Stakeholders identified that several embedded researchers received additional support from Public Health Leaders (Directors of Public Health and Public Health Consultants and Practitioners) who received protected time to undertake their own research in an allied scheme funded by the NIHR (see Kneale et al., 2023). Where both embedded researcher and Public Health Leader roles were present in the same Local Authority, stakeholders reflected on the supportive relationships that formed between the two roles and the joint projects that they were working on. For example, one stakeholder reflected on an unsuccessful funding proposal, and while the outcome was frustrating, the process showed the value of having both posts located within the same organisation:

And [Embedded Researcher] was pivotal in some of that work. [Public Health Leader] led that and [Embedded Researcher] supported with some of our other partners and things. I think without that driving force of [Public Health Leader] and [Embedded Researcher], I’m not quite sure if we would have put in that bid, even though unsuccessful, I think it was a really useful learning exercise.

Although there was significant variation across embedded researcher posts, several stakeholders also reflected on the value of an embedded researchers’ forum which brought these diverse posts together, described as a community of practice by some. This forum was put in place to foster collaboration and learning across all embedded researchers involved in the scheme.

So nationally. All of the [embedded researchers], in the different regions meet. I think it’s every couple of months. So, they have got their own peer support network as well, and they can hear what other people are doing around the region. I think, for them, it’s been an interesting learning curve and widening their horizon and scope of work as well.

#### Expected Impacts and Measures Collected

Enhancing the capacity of research teams was acknowledged by several stakeholders as being a challenging aim to measure and quantify, and similarly, it was acknowledged by some that the capacity for a single role to facilitate such levels of culture change was limited. Instead, some stakeholders offered other indicators of meaningful change that could signal that embedded researchers were influencing a workplace culture that was becoming increasingly research orientated. Such views align with the literature around enlightenment where embedding a research culture might take place through subtle changes in perceptions, conversations and attitudes towards research and evidence, rather than more instrumental and objectively measurable indicators such as the number of research projects and publications:

It’s not necessarily driven by you must have X number of studies, it’s about showing how you can develop those roles to engage a wider remit of people to do research and be interested in research. It’s not necessarily a statistical thing that they’ve got to have done a research study at the end of it.

Stakeholders reflected that the different systems of research within Local Authorities, and the absence of objective metrics that could be used to demonstrate the work of embedded researchers, was viewed as a challenge when making the case for funding or supporting the roles, particularly when contrasted with more established clinical research systems. However, the benefits and importance of these steps towards the embedding of research ideas and processes into organisational cultures was not underestimated by stakeholders, despite the difficulties in measurement. This is demonstrated by one stakeholder when asked to discuss the progress of an embedded researcher that they were co-supervising, where they reflected on a recent presentation about progress delivered to the NIHR, and the comparisons that were made with the work of others in similar roles in clinical settings:

“[Named Practitioner] has done a fantastic presentation, which we’ll get them to present to the rest of the team. But even then, I still think some of the team members look at it and go, “well you haven’t recruited this many numbers of people, and you haven’t set up this many studies, so what is it?”. I still have to champion the need for these sorts of roles in a different way. I think I need to do a bit more of that because we do get questions from some of the team: “what is that role doing? It’s full time. They should be out delivering research in primary care” or “what are they actually doing all day?”. And it’s just a lack of understanding.”

Interviewees also spoke about some of the early changes that were being observed within Local Authorities hosting an embedded researcher that could be integrated into future evaluation frameworks. These included Local Authorities developing and submitting funding proposals to NIHR; Local Authorities and local NIHR networks engaging in promising conversations about supporting research; the formation of networks and hosting meetings between the Local Authority and other research-relevant stakeholders; and the development of research hubs where information on research processes was collated. In terms of funding, many included descriptions of proposals submitted to the HDRC fund. In one case there was a description of research sponsored by the Local Authority being added to the NIHR portfolio. These steps towards research activity were attributed to the embedded researcher being a point of contact between the Local Authority and the NIHR and facilitating links with a broader set of stakeholders including academia, as described by one stakeholder reflecting on the changes enabled by an embedded researcher:

“There isn’t a number. But the I guess the soft measures of that would be things like the fact that we do have in the region a meeting which happens on a regular basis with all the Local Authority public health reps. So that’s established. That’s specifically to talk about research. That’s one aspect. The fact that I’ve been invited to speak at a number of local authorities in the region and with a number of service providers or service leads within the local authority is another measure of that as well, I would say.”

### Findings from Document Analysis

#### Summary of Job Description Features

Table 1 provides an overview of the main points of variation in embedded researchers’ job descriptions. These are described in greater detail below and fuller details are available as supplementary materials.

**Table 1:** Characteristics of job descriptions

Feature	Details	Frequency (*N* = 16)
Advertising organisation	Advertised by LA	3
	Advertised by University	4
	Advertised by NIHR	5
	Advertised by NHS Trust	2
	Not stated	1
	Advertised by ARC	1
Supervision arrangements	LA supervision	5
	Academic supervision	4
	CRN supervision	3
	Not stated	6
	Other supervision	1
Salary	Provided	5
	Not provided	4
	Grade not salary	6
	Secondment - current salary	1
Qualifications	Not stated	4
	Degree level	3
	General postgraduate	6
	MSc	2
	PhD	2
	Professional qualification	3
Specific LA named at the outset	Yes, or Likely	7
	Not named or multiple LAs across a region	9
Aims	NIHR/LA	7
	Local Authority focussed	2
	NIHR focussed	2
	Regional-NIHR	1
	Other	2
	Aims not clearly stated	2

Note. See text for highlights and supplementary materials for full description relating to experience and duties

#### Variation in the Conditions that Embedded Researchers Experienced

Analysis of the 16 available job descriptions for embedded researchers illustrate a large degree of flexibility in the role specifications. Over a quarter of the embedded researcher roles were advertised by universities (*n* = 4) and applied health research collaborations (*n* = 1); a similar number were advertised by the NIHR (*n* = 4); with the remainder advertised by Local Authorities (*n* = 3) or Health Trusts (*n* = 2). An additional role description that had not been advertised externally reflected a set of duties created for an internal candidate within a Local Authority (*n* = 1); a further description was sourced that was used to recruit Local Authorities to express an interest as the basis for creating a further description (*n* = 1). Supervisory arrangements were outlined in ten of the job descriptions, with supervision assumed by managers in Local Authorities (*n* = 5), within universities (*n* = 4), the NIHR (*N* = 3), and the possibility of supervision within the NHS (*n* = 1). Just two descriptions explicitly outlined that supervision and management would take place by individuals across two organisations.

Salaries varied both in terms of how they were described (six provided a grade not a salary), and amount, with the minimum salary advertised amounting to £27,741 and the maximum being up to £42,792 (within a university). Among the descriptions where a salary was provided (*n* = 5), this was set at a standard (National Joint Council) level of £29,631 in three cases. The expectations around highest level of education also varied, with three roles being advertised as open to degree-level qualified researchers with either “*research experience or qualification*”, with the majority requiring some form of (usually unspecified) postgraduate qualification, including a small number of cases where a PhD was described as desirable (*n* = 1) and essential (*n* = 1). In addition to the specifications around qualifications, embedded researchers were expected to demonstrate a considerable degree of technical and substantive expertise, including an understanding of NIHR structures and systems (*n* = 10), knowledge and experience of public health research (*n* = 9), and experience of Local Authority environments and/or public health systems (*n* = 10), with a total of 23 skill areas identified. In terms of the personal attributes, embedded researchers were most frequently expected to have good communication skills (*n* = 10), an ability to work independently (*n* = 10), experience of team working (*n* = 10), and project management skills (*n* = 10), among 15 personal skills identified in the job descriptions.

In the aims contained within some job descriptions, there were indications that embedded researcher roles were assumed to be a form of embedded researcher activity in that they would spend a substantial portion of their time undertaking research-related activities in a setting that may not be their home setting and/or that they were formally affiliated with an academic or research institution (usually the national research funding agency) that was external to where they are usually based. In practice, however, just under half of the role descriptions (*n* = 7) were described as being embedded within a named Local Authority at the outset, with the remainder describing embeddedness across a range of Local Authorities (e.g. Greater Manchester, of which there are ten Local Authorities with public health responsibilities) or broad regions. While all the roles were intended to be embedded within a Local Authority, the analysis suggests that for some embedded researchers, the intervention could also initially involve identification of a Local Authority to become embedded within.

#### Variation in the Aims of Embedded Researcher Posts

Most of the sixteen available job descriptions contained a set of broad aims (*n* = 14), although two contained a description of expected duties or skills required alone. The broad aims of the posts varied in terms of reach and scope. Seven posts described aims that were related to both Local Authority and NIHR concerns jointly. These typically reflected an ambition to increase levels of research activity within Local Authorities and to increase the amount of research supported on the NIHR portfolio, as described in one example:

This is a much-needed opportunity to place network funded staff to support Local Authorities and deliver some evaluation of the effects of such roles in terms of engaging Local Authorities in research (number of Co-Is, recruitment sites, numbers engaging with research training, routine data linkage agreements and facilitation, sourcing and managing evidence and information), as well as the effect on Public Health portfolio development.

In two of the descriptions, the aims were centred around increasing the level of research in Local Authorities and were not explicitly connected to the NIHR, for example “The role will support the development of Public Health research activity in the local authority.”; in another two descriptions, the aims were focussed more on broadening the NIHR portfolio: “The post-holder will play a key facilitating role in developing the portfolio of NIHR studies, normally but not limited to their assigned Clinical Division, across the NIHR”. Finally, another three descriptions contained aims that referred to the development of research cultures across regions, or that supported specific strands of work. Given that only a small proportion of public health research that takes place within the UK is (currently) adopted onto the national research funding agency portfolio of public health research, several of the job descriptions therefore appeared to require, at least in part, what could be considered specialist knowledge of the national research funding agency, its structure, and the type of support it can offer.

All of the embedded researchers were expected to undertake duties that involved the facilitation of research (*n* = 16). The most frequent type of research facilitation activity included making links and forming networks between Local Authorities and the NIHR (*n* = 13), making links and forming networks with academia or public health researchers (*n* = 12), and with various other or undefined stakeholders (*n* = 12). In many cases, the role was described as forming a bridge between Local Authorities and the NIHR; for example, one job description stated:

we expect that the person interested in this role will be employed within Local Authority and be able to dedicate the time to build networks and increase and support research that addresses local priorities, being the conduit between the Clinical Research Network [local NIHR delivery structure] and Local Authority.

Research facilitation was frequently described in amorphous terms, with embedded researchers tasked with promoting research and enhancing a research culture through descriptions such as “Work within the Local Authority to support research to promote health and improve wellbeing”. In total 29 different activities supporting research facilitation were identified, with several occurring within the same job description, illustrating the diversity between roles and the variety of tasks expected to be completed within roles. Nine job descriptions described leading or contributing to research funding proposals as an expected duty, with an additional two requesting that candidates had experience in this area.

The majority of embedded researchers were expected to undertake research production activities (*n* = 15), where we also included descriptions that involved “expanding the NIHR portfolio”. When we excluded research generation that did not involve expanding the NIHR portfolio, this number dropped further (*n* = 4 roles were identified as those where it was expected that research would be generated but not in relation to the NIHR portfolio).

Finally, nine job descriptions included knowledge mobilisation activities to be undertaken including presenting findings (*n* = 4), sourcing evidence (*n* = 4) and using evidence (*n* = 5); for this latter activity, expectations included “support the commissioning of public health research and draw insights from research to support the development and improvement of public health services”. Four job descriptions described producing research for publication within peer-reviewed journals as an expected duty, with an additional two requesting that candidates had experience of publishing within peer-reviewed journals.

Despite the variation in expected characteristics and attributes, we attempted to identify groupings in the job descriptions based on the extent to which the role was intended to meet NIHR objectives (specifically supporting the expansion of Local Authority-based research on the national research funding agency portfolio), and the extent to which the role required knowledge of the national research funding agency, Local Authority systems, and/or public health research. We identified four distinct types of embedded researcher roles:

- NIHR-Local Authority balanced (*n* = 7): These roles require specialist knowledge of both national research funding agency systems and Local Authority public health systems.
- Public Health Research-led (*n* = 4): These roles focus on expanding the national research funding agency portfolio and require knowledge of public health research.
- NIHR-led (*n* = 2): These roles prioritize knowledge of national research funding agency systems over public health research or Local Authority systems.
- Undefined profile (*n* = 3): These roles do not clearly state the desired skill or knowledge profile.

We interpret roles that sought to achieve objectives and required knowledge of both organisations (balanced) involved as evidence of instances where there was a close collaboration in the design of roles; furthermore, we also view the public health research-led roles as being ones where the aims of the role were to bring a synergy between both organisations involved. However, the analysis highlights that in some cases, there was little evidence of collaboration given that the roles reflected the needs of one organisation alone. This is further corroborated in instances where job descriptions contained descriptors that were more aligned with clinical roles including recruiting “patients” for studies (*n* = 2). In one of these, the activities included “involvement in the clinical trials by providing advice and information and acting as the patients advocate”, “if relevant, to be competent in performing clinical tasks required of the protocol, such as vital signs, ECG’s and others”, and “to co-ordinate audits and monitoring visits carried out by pharmaceutical industry regulatory authorities, the MHRA, non-commercial sector research sponsors and Trust R&D staff. Ensuring case report forms and patient notes are prepared in advance”. These clinical duties are not clearly aligned with the work of Local Authority public health teams.

#### Socio-Economic Contexts of Embedded Researcher Roles

Analysis of Office for Health Improvement and Disparities data showed that there was no systematic difference between the deprivation scores, obesity and overweight profiles, and levels of inequalities in life expectancy for males and females respectively between Local Authorities with and without an embedded researcher (see supplementary materials for further information).

#### Understanding the Influence of Embedded Researchers Through Publication and Funding Application Data

Although our analyses of stakeholder interviews above indicate that instrumental measures of research activity may not be suitable for understanding the impact of embedded researchers, such measures were frequently reflected in the job descriptions for the posts and present a common measure of research impact within academia. Here we focus on three sources and indicators: (i) decisions on NIHR funding on the main NIHR funding database where the contracting organisation was a Local Authority; (ii) data from the NIHR portfolio on Public Health studies hosted in Local Authorities adopted by the portfolio; and (iii) data on publication trends of public health studies indexed in PubMed where an author had a Local Authority affiliation. Limited sample size and sparse data present a limitation to these analyses.

With respect to funding proposals, data for instances where the Local Authority was a contracting organisation prior to 2021 (the same year in which the embedded researcher scheme was established) was sparse. Instead, we focussed on 2021 and 2022 and also developed a separate model that incorporates data on HDRC funding decisions (Table 2). In 2021, as the embedded researchers were becoming newly established, there was little perceptible difference in trends based on whether an embedded researcher was in post or not; however, in 2022 Incidence Rate Ratios show that the incidence rate of Local Authorities with an embedded researcher with funding success was three times higher in 2022, and three times higher when we account for HDRC funding. While the results are suggestive, given the short time frame and the lack of data pre-dating the implementation of the embedded researcher scheme, the possibility that these Local Authorities were already more research active cannot be discounted. As we have no baseline data prior to the implementation of embedded researchers, we have not taken this analysis further.

**Table 2:** Negative binomial regression model of funding trends in 2021, 2022

Variable	Model A: project count 2021		Model B: project count 2022		Model C: project count 2022 HDRC	
	Incidence rate ratio	Standard error	Incidence rate ratio	Standard error	Incidence rate ratio	Standard error
Local Authorities with an embedded researcher (vs. those without)	1.020	1.080	3.020	1.709	3.365^**^	1.297
Log base rate	10.354^***^	7.093	1.963	1.777	0.295	0.583
*N*	152		152		152	

Exponentiated coefficients; ***p < 0.0001; **p < 0.001; *p < 0.05

Analysis of national research funding agency (NIHR) portfolio data provides further suggestive evidence that Local Authorities hosting embedded researchers were more active in supporting Public Health research that was hosted on the portfolio. Although most portfolio Public Health research did not involve Local Authorities, descriptive data suggested that those with embedded researchers were more likely to be named sites involved in the conduct of portfolio supported research (Table 3). Furthermore, the only example of Local Authority-led Public Health research hosted on the portfolio was supported by a Local Authority with an embedded researcher.

**Table 3:** Descriptive data on NIHR portfolio hosted research by local authority status

Year	Local authorities with an embedded researcher		Local authorities without an embedded researcher	
	Local authority sponsored research (*Local Authority -led research*)	Academic sponsored research within local authority (*Local Authority -involved in research*)	Local authority sponsored research (*Local Authority -led research*)	Academic sponsored research within local authority (*Local Authority -involved in research*)
Up to and including 2020	0	6	0	5
2021 onwards	1	4	0	0

Finally, we drew on data from PubMed to explore whether we could detect any difference in publication trends between Local Authorities hosting and not hosting an embedded researcher for publications dated between 2018 and 2022. For this outcome therefore, we were able to incorporate data on publication trends at baseline before the implementation of embedded researchers. Overall, we observe a trend where authors based in Local Authorities that had hosted an embedded researcher were consistently much more likely to author or co-author public health research than those that did not host an embedded researcher. A second temporal trend was observed where there was a decrease in the number of studies published by authors with a Local Authority affiliation after 2019 in settings with and without an embedded researcher, a likely reflection of the COVID pandemic and shifting priorities and lower bandwidth to publish papers during a health emergency.

**Fig. 1:**
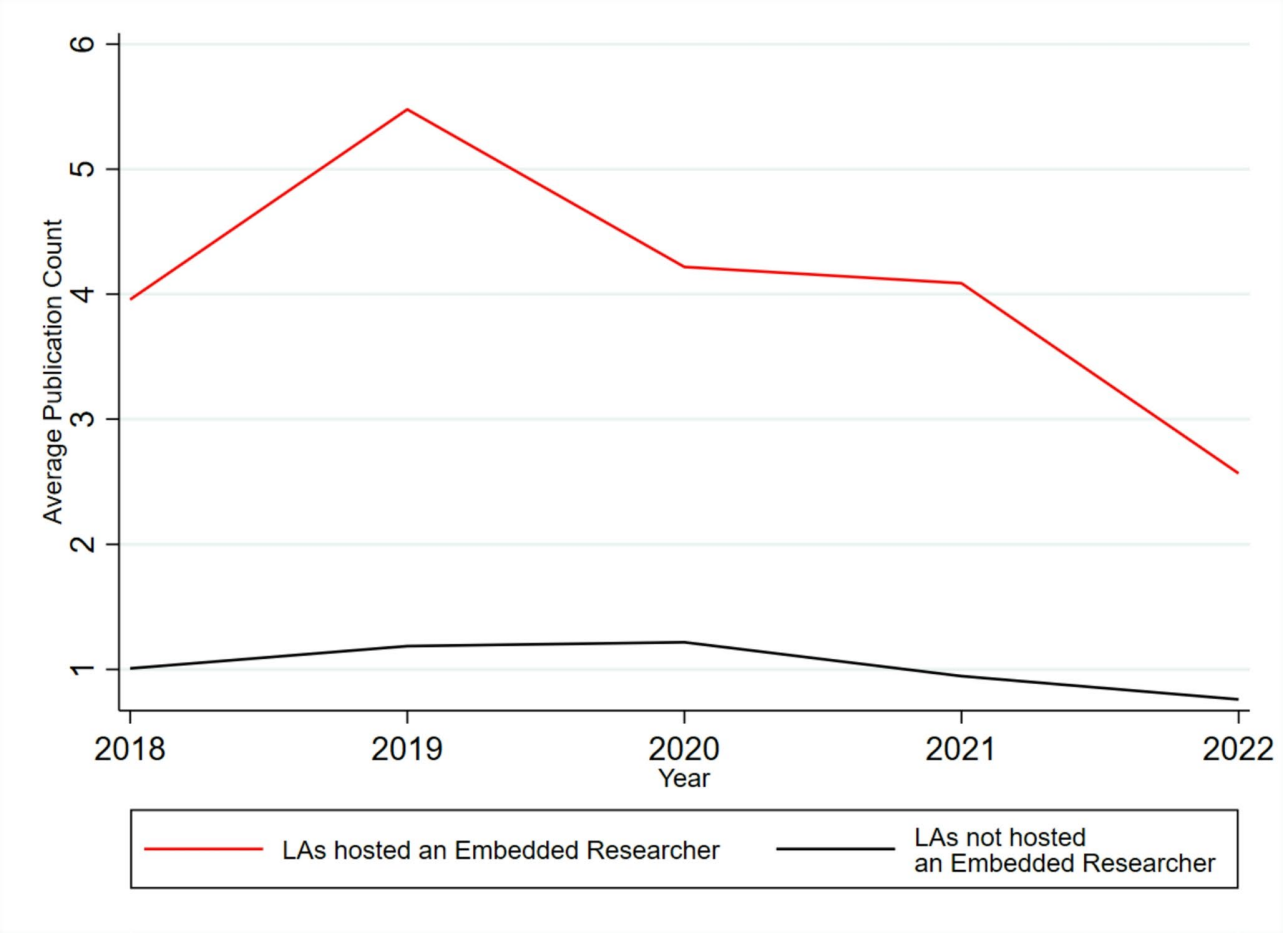
Average publication counts for authors with local authority affiliation by intervention status (hosted an embedded researcher vs. not hosting an embedded researcher)

Longitudinal analysis (Table 4), suggested that the drop in publications being authored by those with a Local Authority affiliation may have been sharper in those Local Authorities that hosted an embedded researcher than those without, although this was not a significant trend. Overall, with respect to publications, the analysis emphasises that those Local Authorities that hosted an embedded researcher were more likely to be research active before the implementation of embedded researchers, but that there was little perceptible impact after implementation of these posts (see Fig. 1).

**Table 4:** Random effects negative binomial regression model of publication trends 2018–2022

Variable	Publication count 2018–2022		Publication count 2018–2022	
	(Embedded researchers) Incidence rate ratio	Standard error	(Embedded researchers and public health leaders) Incidence rate ratio	Standard error
Local Authorities with an Embedded Researcher (vs. those without)	0.778	0.112	0.781	0.108
Number of groups (observations per group)	152 (5)		152 (5)	

***p < 0.0001; **p < 0.001; *p < 0.05

## Discussion

This paper explores how embedded researcher roles are set-up, the extent to which they represent jointly designed and implemented roles, and how far the expectations around embedded researcher roles can be evaluated. Embedded researcher models are becoming increasingly popular across a range of sectors as a potential strategy for bridging the gap between generators and consumers of research (Kneale et al., 2024; Ward et al., 2021). However, the multiple diverse organisations that are inherently involved with these roles adds a high degree of complexity to their design.

Our evidence indicated that levels of collaboration between research and practice organisations varied substantially in the design and implementation of embedded researcher roles across the NIHR-Local Authority partnerships involved in our case study. Our findings indicate that achieving a strong level of engagement and collaboration in the design and implementation of embedded researcher posts can be challenging and was not always achieved. Recruitment challenges partly due to COVID-19 and Local Authority capacity constraints meant the NIHR received little engagement from Local Authorities in some cases. Furthermore, the evidence here indicates that pre-existing relationships between Local Authorities and the national research funding agency/universities were patchy. Part of this may reflect the historically clinical focus of the NIHR, with the embedded researcher roles intended facilitate the national research funding agency to better understand public health decision-making cultures.

We identify three ‘downstream’ implications of the challenges experienced in the setting-up and implementation of the roles below, which include: inconsistent alignment of roles with the needs of practice organisations, inappropriate evaluation metrics, and unrealistic expectations.

First, while our interviews emphasised that roles had been designed flexibly to prioritise the needs of the Local Authority, these priorities were not clearly reflected in several job descriptions, with a stronger emphasis on NIHR-based objectives. In such cases, the aims of embedded researcher positions appeared to be misaligned with what might be needed in practice. This included one instance where the embedded researcher role was seemingly advertised as a Clinical Nursing position with expected duties matching those of individuals who run clinical trials; such instances could be considered the product of poor collaboration between participating partners in the development of an embedded researcher role. Some stakeholders also demonstrated comparatively low levels of knowledge about Local Authority needs, and job descriptions were lacking in specificity on capacity building objectives.

Misalignment and a failure to adequately reflect the needs of the policy/practice partner also had implications for the evaluation of the posts. Evaluation measures could reflect highly instrumental outcomes of change in research activity (e.g., journal publications) which, although common measures of success within research institutions such as the NIHR, are unlikely to effectively capture the shifts embedded researchers can be expected to activate within Local Authority public health teams. Indeed, this research highlighted how ‘downstream’ or ‘instrumental’ quantitative measures of research activity alone are unlikely to capture the changes brought about by embedded researchers. We observed that Local Authorities involved in the embedded researcher scheme were more likely to obtain funding than those without, and the only instance of Local Authority sponsored research to successfully attain support from the NIHR through its portfolio of studies was one that hosted an embedded researcher. However, we were unable to discount the possibility that these Local Authorities may have been more engaged in applying for funding before the intervention started. Indeed, analysis of publication data suggests that Local Authorities that hosted an embedded researcher were already among the most research active with respect to authoring public health studies and the analyses indicate that embedded researchers did not influence publication trends, albeit with several caveats outlined below. Despite suggestive evidence, we therefore remain unable to ascertain the impact of embedded researchers using only these academic-oriented instrumental measures.

As we outline in our introduction, embedded researchers are aligned with a movement towards facilitating the mobilisation of evidence to inform public health decision-making. However, knowledge mobilisation – for example in sourcing evidence for decision-making or presenting research findings – is not represented in the expected duties of many embedded researcher posts (just over half described knowledge mobilisation duties). While we acknowledge that there are overlaps between knowledge mobilisation duties and other areas (e.g. research facilitation), this does appear to be something of an anomaly and perhaps suggests a need to further theorise what can be achieved within embedded researcher roles and their contribution to creating research active environments. Research active environments should be concerned with judiciously utilising existing relevant research as much as they are concerned with generating new research. Furthermore, accounts from embedded researchers themselves emphasise that they play an important role in knowledge mobilisation, and some of the important changes that are observed through their work involve this area of research activity (Kneale et al., 2023).

Our results in this study underline the importance of adopting evaluation approaches that can capture a breadth of changes in research activity influenced by embedded researchers, including evidence around the role of research in enlightenment, and the importance of qualitative methods for capturing these incremental changes. Reassuringly, the misalignment between objective measures of change in research activity with nuanced, gradual shifts was acknowledged within stakeholder interviews among those involved in designing, implementing or managing the embedded researcher scheme. The interview data also highlight some of the important incremental changes in research activity that embedded researchers helped to generate, including new conversations between organisations and greater embeddedness of Local Authorities within research networks; these can represent important pre-cursors to organisations making more systemic changes and becoming more embedded within local research infrastructures. Nevertheless, such qualitative metrics were not always well integrated within job descriptions which could hinder embedded researchers in understanding and measuring their own progress.

Some of these ‘softer’ measures of the influence of embedded researchers could be collected through instrumental metrics – for example in terms of number of presentations or number of meetings. However, such measures tend to be uninformative without accompanying contextual detail. Evaluating the impact of embedded researcher roles therefore calls for qualitative approaches, either as standalone methods or in combination with quantitative ones. For example, our own efforts to understand embedded researcher work highlighted the value of diary methods. Elsewhere, there is a growing body of autoethnographic studies that document and reflect on the work of embedded researchers (Kneale et al., 2024). These approaches could be developed further, pointing to the need for more creative and innovative methods that can account for the subtle, relational, and cultural shifts that embedded researchers are working to influence.

Finally, some embedded researcher roles were created with aims that were not only misaligned with Local Authority priorities as described above, but also unrealistic and poorly articulated. Posts were operationalised with a set of aims and expectations that were not always clear and commensurate with what was possible for a single individual to achieve. While there may have been tacit recognition of this among those involved in designing and managing the scheme, this was not always communicated clearly to embedded researchers themselves, who not only spent considerable time trying to make sense of the expectations of their role, but also felt a considerable degree of stress in trying to fulfil these broad and inappropriate expectations in some cases (Kneale et al., 2023). While this was by no means the dominant experience of the role, which was viewed by many as rewarding and fulfilling, it does nevertheless suggest that a more considered co-designed approach to planning the roles that considers the context in which embedded researchers are to be placed could pay dividends.

### Implications

The aforementioned challenges and research published elsewhere underscores the importance of co-designing embedded researcher interventions (Cheetham et al., 2018; Kneale et al., 2023). However, this research also revealed some potential challenges that can occur when embedded researcher roles are highly varied and tailored to the local context. Our document analysis highlighted significant variation across several dimensions of embedded researcher roles. These included expected qualifications, management arrangements, embeddedness, activities, and aims. To some extent, this variation reflects how, in some cases, the roles were designed with the needs of Local Authorities in mind. However, the degree of variation around salaries was unexpected, and suggests that some embedded researchers were entering the role on an unequal basis compared with their peers. Similarly, further inequity is created when additional support structures, such as the Public Health Leaders, are put in place in some Local Authorities but not others. Significant variation can also create confusion across those undertaking the roles simultaneously across different organisations, particularly where embedded researchers collectively discuss their work and progress. As such, any variation across roles should be clearly articulated to embedded researchers from the outset.

A second implication of co-designing embedded researcher roles is that it is likely to result in a self-selecting process for recruiting practice organisations, as was the case in this research. In our case study, self-selection resulted in embedded researchers being (i) *not* necessarily being placed in those Local Authorities where there were the greatest public health needs and where a more research active culture might lead to the largest gains; and instead (ii) being situated within Local Authorities that may already have had some elements of a research active culture. The implications of self-selection, and acknowledging that different Local Authorities may be more well placed than others to engage in co-design of embedded researchers’ roles, may be important factors to consider in the design and management of future embedded researcher schemes.

### Limitations

We note limitations surrounding this research. Firstly, job descriptions were not obtained for all posts, and contextualising interviews were only conducted with a minority of local NIHR structures. Secondly, this research started after the implementation of embedded researchers for the most part, and with the possible exception of publication trends as a singular indicator, we were unable to identify suitable measures or data to ascertain the baseline levels of research activity prior to the implementation of embedded researchers. In addition, while the quantitative analyses included the vast majority of LAs in England with public health responsibilities, this nevertheless represents a relatively small sample size, and the quantitative analyses are likely to have been underpowered. Thirdly, the document analysis presents an analysis of static documents, and expectations around how embedded researcher roles were to be operationalised may not be fully reflected in the documents analysed here. Fourth, while we have acknowledged earlier that any differences in research funding and publications may be due to differences pre-dating the implementation of embedded researchers, we also acknowledge that success with either funding or publications can take years to emerge, and that any differences may emerge only after a substantial period of time has passed.

## Conclusions

The challenge of bridging the gap between research and policy, particularly within public health where research evidence may be competing with political interests in influencing decisions, is immense and often requires large scale intervention (see, for example, Van Der Graaf et al. (2020). Embedded researchers are a promising intervention that could help capitalise on an appetite among Local Authority public health teams to incorporate research evidence into decision-making (Cheetham et al., 2023; Vindrola-Padros et al., 2017). Our results underscore the importance of designing a collaborative research agenda in establishing similar embedded researcher roles in the future. Within the process of embedding researchers into host organisations, there exists a stage before a researcher is placed within a host setting in which co-designing and tailoring of the role take place (Kneale et al., 2023). The present work presents one of the first studies to explicitly explore the setting up of embedded researcher posts and the downstream implications of variation in implementation. We found that the success of these collaborative processes is moderated by the extent and quality of pre-existing relationships between the host and home/sending organisations and existing capacity of hosting organisations. We suggest that, given its many potential effects on the implementation of embedded researcher posts, this stage is not something that should be rushed. Rather, time should be afforded for interorganisational learning and cultivating mutually beneficial relationships across research and practice organisations.

## Electronic Supplementary Material

Below is the link to the electronic supplementary material.

Supplementary Material 1

## Data Availability

No datasets were generated or analysed during the current study.
