# Supplementary material for: Embedding Researchers into Local Government Public Health Teams: Exploring Co-Design, Implementation and Evaluation Challenges Through Document and Contextual Analysis
Source: Glob Implement Res Appl. 2025 Sep 3;5(4):474–88. doi: 10.1007/s43477-025-00179-1 (PMC12638368; doi:10.1007/s43477-025-00179-1)
Supplement: Supplementary file 1 — Supplementary Material 1 [file 43477_2025_179_MOESM1_ESM.docx]

# Supplementary materials

**Table 1**

Summary of themes, sub-themes and codes from stakeholder interviews

| Theme | Sub-theme | Code(s) | Frequency (N=7) |
| --- | --- | --- | --- |
| Challenges in Collaboration and Implementation | COVID-19 related challenges to working together | COVID-19 challenges | 7 |
|  | Collaboration challenges on the recruitment of roles | Recruitment challenges | 5 |
|  |  | Recruitment inequities or competition | 4 |
|  |  | Recruitment process CRN driven (lack of collaboration) | 3 |
|  |  | Other recruitment challenges: unusual relationships | <3 respondents |
|  | Collaboration challenges on the design of roles | Design of posts CRN-led | 4 |
|  |  | Design based on previous posts | 4 |
|  |  | Other recruitment challenges: Design of role not integrated into needs of broader CRN/NIHR; Challenges in understanding role or purpose | Each <3 respondents |
|  | Collaboration challenges and facilitators in provision of oversight and support for embedded researchers | Flexibility in set-up and oversight | 7 |
|  |  | Community of practice | 6 |
|  |  | Lack of oversight | 5 |
|  |  | Light-touch oversight | 4 |
|  |  | Patchy or inefficient relationships in oversight | 3 |
|  |  | Other oversight challenges: Clarity in oversight arrangements; Oversight not joined up with parallel scheme | Each <3 respondents |
|  | Other collaboration challenges | Anticipated changes in NIHR structures; Other NIHR activities occurring | All <3 respondents |
| Setting and evaluating expectations and aims for embedded researchers | Aims of roles as articulated | Increasing research capacity and/or activity | 5 |
|  |  | Aims challenging to measure | 5 |
|  |  | CRN focussed aims | 4 |
|  |  | Broad aims articulated | 4 |
|  |  | Aims focussed around linking | 4 |
|  |  | Aims or purpose described as unclear | 3 |
|  |  | Aim to make research more visible | 3 |
|  |  | Other codes for aims of roles: Support research of others; Understand potential of role as pilot; Low expectations | All <3 respondents |
|  | Expected impacts and measures collected | Perceived barrier to understanding role: clinical-public health divide | 4 |
|  |  | Perceived barrier to understanding role: lack of research culture | 4 |
|  |  | Perceived facilitator: consultant post being established at the same time | 5 |
|  |  | Impacts already visible | 4 |
|  |  | Perceived barrier to understanding role: job or funding insecurity | 3 |
|  |  | Other codes around expected impacts and measures collected: Impacts unclear; Operational measures only collected; A single person can’t achieve everything; Perceived barrier – LAs are complex organisations; Eyes and Ears on the ground | All <3 respondents |
| LA: Local Authority (LAs: Local Authorities); NIHR: National Institute of Health and Care Research; CRN: Clinical Research Network | | | |

**Table 2**

Extended information on characteristics of job descriptions

| Feature | Details | Frequency (N=16) |
| --- | --- | --- |
| Advertising organisation | Advertised by LA | 3 |
|  | Advertised by University | 4 |
|  | Advertised by NIHR | 5 |
|  | Advertised by NHS Trust | 2 |
|  | Not stated | 1 |
|  | Advertised by ARC | 1 |
| Supervision arrangements | LA supervision | 5 |
|  | Academic supervison | 4 |
|  | CRN supervision | 3 |
|  | Not stated | 6 |
|  | Other supervision | 1 |
| Salary | Provided | 5 |
|  | Not provided | 4 |
|  | Grade not salary | 6 |
|  | Secondment - current salary | 1 |
| Qualifications | Not stated | 4 |
|  | Degree level | 3 |
|  | General postgraduate | 6 |
|  | MSc | 2 |
|  | PhD | 2 |
|  | Professional qualification | 3 |
|  | Not clearly stated | 4 |
| Experience - technical | Knowledge of research design | 7 |
|  | Understanding of NIHR | 10 |
|  | Knowledge of PH Research | 9 |
|  | Knowledge of statistical/database software | 5 |
|  | Awareness of GDPR etc | 6 |
|  | Data/research governance and ethics | 6 |
|  | Quantitative data collection/sources/analysis | 5 |
|  | Data quality and data management | 6 |
|  | Experience of LA environment/ PH systems | 8 |
|  | Publication experience | 3 |
|  | Teaching experience | 3 |
|  | Experience of multi-agency working | 1 |
|  | Generic software | 8 |
|  | Knowledge mobilisation | 1 |
|  | Clinical research/trials skills/experience | 2 |
|  | Experience of evaluation | 1 |
|  | Practitioner experience | 1 |
|  | Organisational audit | 1 |
|  | PPI experience/skills | 1 |
|  | Data interpretation skills | 1 |
|  | School environment research | 1 |
|  | Evidence usage skills | 1 |
|  | Experience of applying for funding | 1 |
| Experience - personal | Communication skills | 10 |
|  | Ability to work idependently | 10 |
|  | Flexibility | 3 |
|  | Organisational skills | 9 |
|  | Team work | 10 |
|  | Presentation skills | 6 |
|  | Ability to travel | 7 |
|  | Project management | 10 |
|  | Writing skills | 6 |
|  | Other | 5 |
|  | Interpersonal skills | 8 |
|  | Understanding of equality/diversity issues | 2 |
|  | Creative/strategic thinking | 2 |
|  | Leadership/management of staff | 2 |
|  | Concentration skills and focus | 2 |
| Specific LA named at the outset | Yes or Likely | 7 |
|  | Not named or multiple LAs across a region | 9 |
| Aims | NIHR/LA | 7 |
|  | Local Authority focussed | 2 |
|  | NIHR focussed | 2 |
|  | Regional-NIHR | 1 |
|  | Other | 2 |
|  | Aims not clearly stated | 2 |
| Research Production Duties | Qualitative data collection | 2 |
|  | Qualitative data analysis | 2 |
|  | Evidence synthesis/Literature review | 2 |
|  | CRN PH Portfolio Research | 11 |
|  | Quantitative data analysis | 3 |
|  | Quantitative data collection | 3 |
|  | Data collection | 2 |
|  | Research planning/assessment of feasibility | 3 |
|  | Producing research (method unspecified) | 4 |
| Research facilitation activities | Data entry | 3 |
|  | Supporting research - design | 9 |
|  | Teaching/training | 8 |
|  | Data preparation/database management | 7 |
|  | Making links with academia and/or PH researchers | 12 |
|  | Making links with NIHR/fulfilling NIHR objectives | 13 |
|  | Workforce development (high level) | 10 |
|  | Making links within LA | 8 |
|  | Troubleshoot data issues | 1 |
|  | Assist with funding applications | 9 |
|  | Creating, developing or supporting res governance | 6 |
|  | Making links with other/undefined stakeholders | 12 |
|  | Promoting research/research culture | 11 |
|  | Support communities of practice | 2 |
|  | Identifying research priorities | 8 |
|  | Focussing on underrepresented groups | 1 |
|  | Personal development | 8 |
|  | Guidance and advice on research | 6 |
|  | Manage/performance/quality manage research | 5 |
|  | Making links/communicating with public | 2 |
|  | Supporting research - recruitment | 4 |
|  | Co-supervise students | 1 |
|  | Activities to support identification of res needs | 1 |
|  | Manage PH Steering committee | 1 |
|  | Cover other LA duties | 1 |
|  | Supporting research - commissioning | 1 |
|  | Managing staff | 1 |
|  | Budgetary responsibilities | 1 |
| Knowledge mobilisation activities | Presenting findings | 4 |
|  | Publishing peer reviewed literature | 4 |
|  | Sourcing evidence | 4 |
|  | Using evidence | 5 |
|  | Sharing information | 3 |

**Table 3**

Characteristics of Local Authorities with and without PHLARPs

|  | Local Authorities without a Research Practitioner | | Local Authorities with a Research Practitioner | |  |
| --- | --- | --- | --- | --- | --- |
|  | Sample Size | Mean | Sample Size | Mean | Difference in Means |
| Mean Index of Multiple Deprivation Score | 129 | 22.704 | 23 | 24.233 | 1.53 |
| Mean Proportion of Obese Adults in Population | 127 | 9.864 | 23 | 9.887 | 0.022 |
| Index of inequality in life expectancy at birth for females | 127 | 7.162 | 23 | 6.661 | 0.502 |
| Index of inequality in life expectancy at birth for males | 127 | 8.696 | 23 | 9.252 | 0.557 |
| *Note: sample size differ where there are no available data for certain LAs published by OHID; *Asterisks indicate statistically significant difference* | | | | | |
